# Correlation of Endoscopic and Histopathological Diagnoses in Upper Gastrointestinal Tract Lesions: A Cross-Sectional Study

**DOI:** 10.7759/cureus.69553

**Published:** 2024-09-16

**Authors:** Sudhasmita Rauta, Pratima Baisakh, Aswini K Sahoo, Dhiren K Panda, Manas R Baisakh, Sushree s Dash

**Affiliations:** 1 Pathology, Hi-Tech Medical College and Hospital, Bhubaneswar, IND; 2 Anatomy, Institute of Medical Sciences and SUM Hospital, Siksha 'O' Anusandhan Deemed to be University, Bhubaneswar, IND; 3 Internal Medicine, Institute of Medical Sciences and SUM Hospital, Siksha 'O' Anusandhan Deemed to be University, Bhubaneswar, IND; 4 Histopathology, Prolife Diagnostics, Bhubaneswar, IND; 5 Pathology and Laboratory Medicine, Prolife Diagnostics, Bhubaneswar, IND

**Keywords:** diagnostic accuracy, duodenitis, endoscopy, esophagitis, gastritis, histopathology, neoplastic lesions, non-neoplastic lesions, upper gastrointestinal tract

## Abstract

Introduction

Gastrointestinal tract (GIT) endoscopy with biopsy is essential for diagnosing and managing various GIT diseases, including malignancies and inflammatory conditions. This study aimed to analyze the spectrum of histopathological lesions in the GIT and their correlation with the endoscopic findings.

Methodology

This retrospective observational study was conducted at a tertiary medical college involving 114 patients who underwent GIT endoscopy between June 2023 and June 2024. This study focused on lesion types across different GIT sites (esophagus, stomach, and duodenum) and the agreement between endoscopic and histological diagnoses.

Results

Lesions were most prevalent in the stomach (52.6% of cases, n=60), followed by the duodenum (35.1%, n=40) and esophagus (12.3%, n=14). Significant correlations were found between endoscopic and histological diagnoses in the esophagus (concordance for esophagitis was 100%) and stomach (concordance for gastritis was 95%). In the duodenum, the concordance was high for duodenitis (100%) but lower for other lesions such as neuroendocrine tumors (71.43%). However, Cohen's kappa values indicated poor overall agreement across all sites (κ=0.49), reflecting variability in diagnostic accuracy.

Conclusion

This study highlights the reliability of endoscopic procedures for diagnosing upper GIT lesions, particularly in the esophagus and stomach, while emphasizing the challenges in diagnosing duodenal lesions. These findings support the need for targeted screening and further research to enhance diagnostic accuracy and patient outcomes in GIT diseases.

## Introduction

Gastrointestinal tract (GIT) endoscopy coupled with biopsy plays a pivotal role in diagnosing and managing various GIT diseases, including malignancies and inflammatory conditions [1-2]. This procedure is particularly crucial in identifying dyspepsia, gastroesophageal reflux disease (GERD), Barrett’s esophagus, and esophageal and stomach carcinomas [1]. Endoscopic biopsy allows for direct visualization and targeted sampling of suspicious areas, enabling the early detection and accurate diagnosis of pathological conditions. Histopathological examination of biopsy samples obtained during endoscopy offers vital insights into the nature and extent of GIT conditions, thereby guiding clinical decision-making and treatment strategies [2]. Liquid biopsies are emerging as promising tools for early cancer detection and monitoring, thereby enhancing patient outcomes in GIT cancers [3].

The GIT presents a spectrum of disorders ranging from benign inflammatory conditions to malignant neoplasms, with dyspepsia and GERD being common indications for upper GIT endoscopy [4-6]. Dyspepsia, characterized by upper abdominal pain or discomfort, can signify various underlying issues such as peptic ulcer disease, gastritis, or gastric cancer [5]. In contrast, GERD involves reflux of stomach acid into the esophagus, leading to symptoms such as heartburn and regurgitation, potentially progressing to Barrett’s esophagus and increasing the risk of esophageal adenocarcinoma [2]. Endoscopic techniques play a crucial role in diagnosing and managing these conditions, aiding the early detection of malignancies and guiding appropriate patient management [4,6].

Esophageal and gastric carcinomas pose significant global health challenges with high morbidity and mortality rates. The early detection of these cancers is paramount for improving patient outcomes. Esophageal carcinoma typically manifests as either squamous cell carcinoma or adenocarcinoma, each characterized by distinct histopathological features and etiological factors [7,8]. In contrast, gastric carcinoma is often diagnosed at advanced stages, underscoring the importance of comprehensive histopathological assessment to determine its type, grade, and stage, which is crucial for devising appropriate therapeutic strategies [7,9]. Endoscopic techniques coupled with biopsy play a pivotal role in the early detection and accurate diagnosis of both esophageal and gastric carcinomas, enabling timely interventions and improved prognosis [10,11,12].

The primary aim of this study was to determine the spectrum of histopathological lesions of the GIT and compare these findings with endoscopic observations. This involved a detailed analysis of demographic data (age and sex distribution), the types of lesions identified, and the correlation between endoscopic and histological diagnoses across different GIT sites, including the esophagus, stomach, and duodenum.

## Materials and methods

Study design

This study employed a retrospective observational design to analyze histopathological lesions of the GIT and their correlation with endoscopic findings.

Sample selection

The study cohort consisted of 114 patients from a tertiary medical college and hospital who underwent gastrointestinal endoscopy over one year, from June 2023 to June 2024. Biopsy samples were collected from lesions observed during these procedures at different GIT sites (esophagus, stomach, and duodenum). The tissue samples were processed for histopathological investigation following Hegazy’s simplified method of tissue processing, which consumes less time and chemicals [13]. The histopathological examination involved standard stains such as hematoxylin and eosin (H&E) for general morphology, periodic acid-Schiff for mucin detection, and Giemsa for identifying *Helicobacter pylori*, ensuring comprehensive evaluation of the biopsied tissues.

Data collection

Demographic Data

Demographic information, including age and sex distribution, was extracted from electronic medical records and endoscopy reports.

Endoscopic Examination

**Figure 1:**
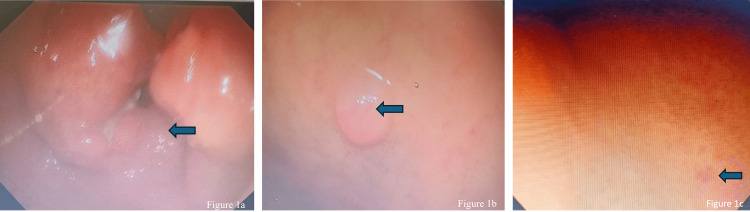
Endoscopic images of gastrointestinal lesions 1a shows circumferential ulceronodular growth observed on the gastric antrum, 1b shows submucosal swelling in the duodenum, and 1c shows Erosion in the gastric corpus.

Endoscopic procedures were performed by a gastroenterologist using standard diagnostic protocols. Biopsy samples were obtained during endoscopy from identified lesions in the duodenum, esophagus, and stomach. Endoscopic images (Figure 1) captured during these procedures provided essential visual evidence of the lesion characteristics and locations, which were subsequently correlated with histopathological findings.

Histopathological Analysis

**Figure 2:**
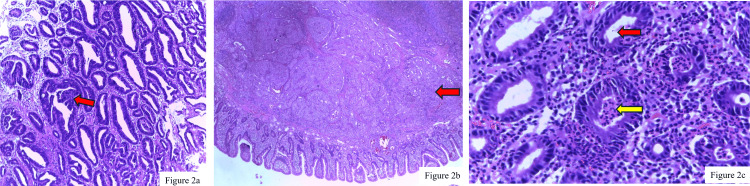
Histopathological examination of gastrointestinal biopsy samples 2a: Adenocarcinoma of the stomach characterized by atypical tubular and branching glands infiltrating the muscularis mucosae (H&E staining, 400x magnification). The red arrow indicates the area of infiltration. 2b: Neuroendocrine tumor of the duodenum composed of round to oval tumor cells with eosinophilic cytoplasm and granular chromatin, arranged in lobular nests indicated in red arrow (H&E staining, 100x magnification). c: Severe chronic active gastritis with frequent foveolitis and foveolar abscess formation (H&E staining, 400x magnification). The red arrow highlights gastric foveolitis, and the yellow arrow indicates foveolar abscess formation, revealing severe activity. H&E: hematoxylin and eosin

Histopathological analysis of the biopsy samples was performed by pathologists following established protocols for tissue processing and staining. Microscopic examination of these samples (Figure 2) provided detailed insights into the nature of the lesions, ranging from malignancies such as adenocarcinoma (Figure 2a) to benign conditions such as chronic active gastritis (Figure 2c).

Data analysis

The data were analyzed to determine the age-, site-, and sex-wise distribution of the lesions in the upper GIT. Statistical analysis was performed using the chi-square test to evaluate the correlation between endoscopic findings and histopathological diagnoses. Statistical significance was set at p<0.05 using SPSS Statistics version 25 (IBM Corp. Released 2017. IBM SPSS Statistics for Windows, Version 25.0. Armonk, NY: IBM Corp.).

The results were compared with those in the existing literature to validate the findings and understand the diagnostic accuracy of endoscopy for upper GIT lesions. References to relevant studies were made to contextualize the results and highlight the importance of histopathological confirmation in the diagnosis of upper GIT diseases.

Ethical considerations

The study protocol was approved by the Ethical Committee of the Institute of Medical Sciences and SUM Hospital (approval number: IMS/IEC/107/2023). Informed consent was waived due to the retrospective nature of the study, and patient data were anonymized to protect confidentiality.

Limitations

Limitations included the retrospective study design, potential biases in patient selection, and reliance on existing medical records, which may have affected data completeness and accuracy.

## Results

Demographics and patient characteristics

**Figure 3:**
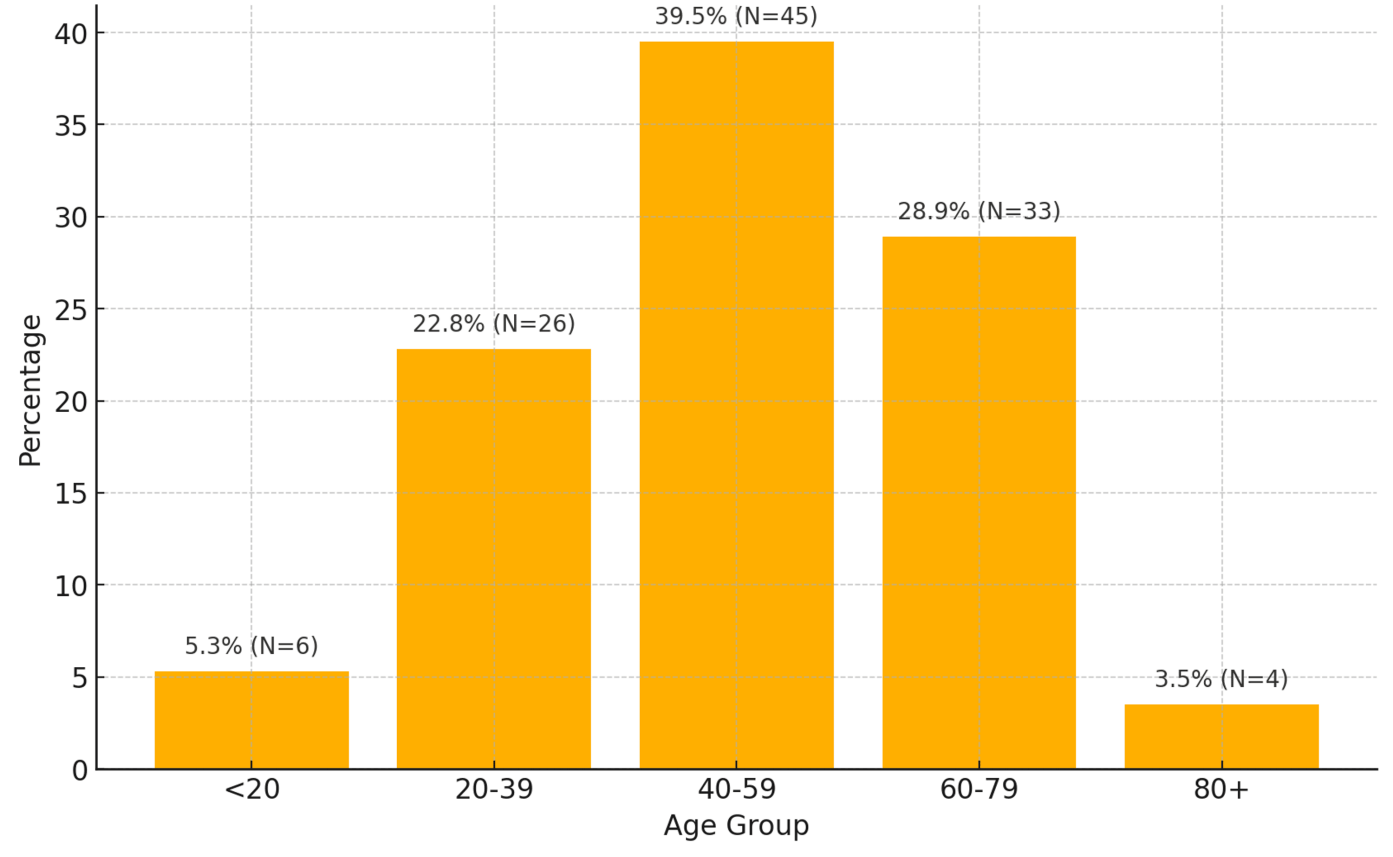
Age distribution of patients The chart shows the age distribution of patients with age ranges on the x-axis (<20, 20-39, 40-59, 60-79, 80+ years) and percentages on the y-axis. The highest percentage was in the 40-59 age group (39.5%, N=45).

The mean age was 52.25 years (SD=18.40), ranging from four to 94 years, and an IQR indicated that 50% of the patients were aged between 42 and 65 years. The age distribution showed that the majority of patients (39.5%, N=45) were between 40 and 59 years, followed by those aged 60 and 79 years (28.9%, N=33) (Figure 3).

**Figure 4:**
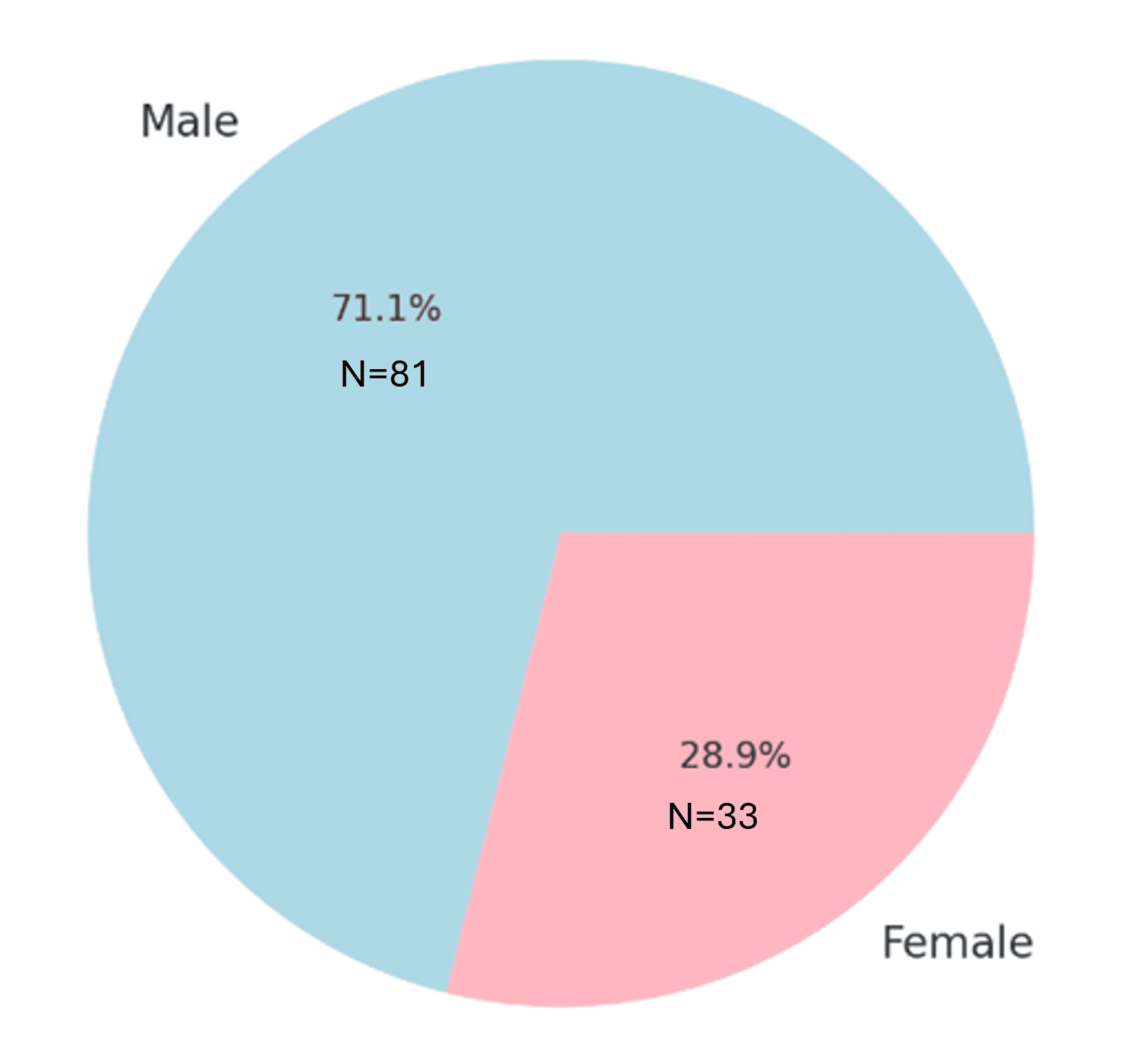
Gender distribution of patients The gender distribution in a pie chart with percentages and N numbers for both males and females.

The sex distribution was skewed toward males, comprising 71.1% (81 patients) of the cohort, whereas females comprised 28.9% (33 patients) (Figure 4).

Lesion distribution across GIT sites

**Figure 5:**
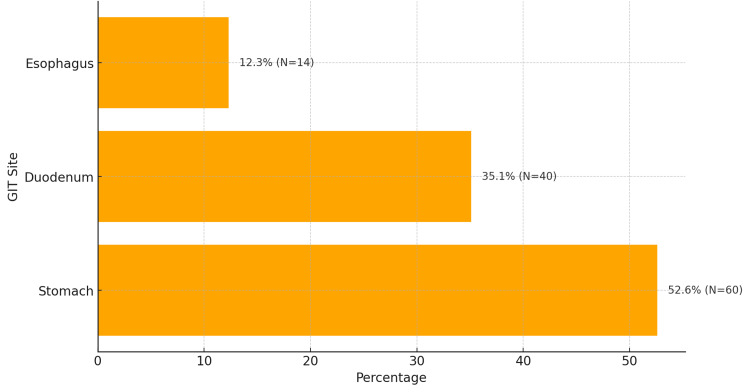
Lesion distribution across GIT sites The distribution of lesions across various GIT sites with percentages and N numbers. GIT: gastrointestinal tract

Lesions were observed across various GIT sites, with the stomach being the most prevalent site (52.6% of cases, N=60), followed by the duodenum (35.1%, N=40) and esophagus (12.3%, N=14) (Figure 5).

Concordance between endoscopic and histopathological diagnoses

This study examined the agreement between endoscopic and histopathological diagnoses by using cross-tabulation and Cohen’s kappa analysis.

Duodenum

The endoscopic diagnosis of adenocarcinoma in the duodenum had an 83.33% (N=10) concordance with the histopathological findings. Duodenitis exhibited perfect agreement at 100% (N=12), whereas neuroendocrine tumors showed 71.43% (N=5) concordance. Coeliac disease and lymphangiectasia also displayed strong concordance rates, reflecting the reliability of endoscopy under certain conditions (Table 1).

**Table 1:** Duodenum concordance table

Endoscopic finding	Adenocarcinoma (%) (N)	Coeliac disease (%) (N)	Duodenitis (%) (N)	Lymphangiectasia (%) (N)	Neuroendocrine tumor (%) (N)
Adenocarcinoma	83.33 (N=10)	0 (N=0)	16.67 (N=2)	0 (N=0)	0 (N=0)
Coeliac disease	0 (N=0)	33.33 (N=2)	66.67 (N=4)	0 (N=0)	0 (N=0)
Duodenitis	0 (N=0)	0 (N=0)	100 (N=12)	0 (N=0)	0 (N=0)
Lymphangiectasia	0 (N=0)	0 (N=0)	0 (N=0)	100 (N=3)	0 (N=0)
Neuroendocrine tumor	0 (N=0)	0 (N=0)	14.29 (N=1)	0 (N=0)	71.43 (N=5)

Esophagus

The concordance rate for esophageal carcinoma was 57.14% (N=8), indicating a moderate agreement. Barrett’s esophagus had a lower concordance rate of 25% (N=3), suggesting significant diagnostic challenges. However, esophagitis showed perfect concordance of 100% (N=9), underscoring the effectiveness of endoscopy for this condition (Table 2).

**Table 2:** Esophagus concordance table

Endoscopic finding	Carcinoma (%) (N)	Barrett's esophagus (%) (N)	Esophagitis (%) (N)
Barrett's esophagus	0 (N=0)	25 (N=3)	75 (N=9)
Carcinoma	85.72 (N=12)	0 (N=0)	14.29 (N=2)
Esophagitis	0 (N=0)	0 (N=0)	100 (N=9)

Stomach

Gastric adenocarcinoma showed a concordance rate of 78.26% (N=18), whereas gastritis showed a high concordance rate of 95% (N=38). Polyps were accurately diagnosed with 100% (N=5) concordance, and ulcers had an 80% (N=8) concordance rate (Table 3).

**Table 3:** Stomach concordance table

Endoscopic finding	Adenocarcinoma (%) (N)	Gastritis (%) (N)	Polyp (%) (N)	Ulcer (%) (N)
Adenocarcinoma	78.26 (N=18)	21.74 (N=5)	0 (N=0)	0 (N=0)
Gastritis	0 (N=0)	95 (N=38)	0 (N=0)	0 (N=0)
Polyp	0 (N=0)	0 (N=0)	100 (N=5)	0 (N=0)
Ulcer	0 (N=0)	0 (N=0)	0 (N=0)	80 (N=8)

Cohen's kappa analysis

Cohen's kappa score across all sites was 0.49, indicating a moderate level of agreement between the endoscopic and histopathological diagnoses. This score reflects the degree of consistency between the diagnostic outcomes of the two methods.

## Discussion

This study provides critical insights into the diagnostic concordance between endoscopic and histopathological findings in patients with upper GIT lesions. Although endoscopy is the primary diagnostic tool for GIT pathologies, our findings highlight the importance of histopathological confirmation to ensure diagnostic accuracy.

Diagnostic concordance

The overall Cohen's kappa score of 0.49 reflects a moderate agreement between endoscopic and histopathological diagnoses. This level of concordance suggests that despite its utility, endoscopy is not entirely reliable as a standard diagnostic method. Specifically, the study demonstrated high concordance for certain conditions, such as adenocarcinoma in the duodenum (83.33%) (Table 1) and gastritis in the stomach (95.0%) (Table 3), indicating that endoscopy can effectively identify specific lesions. However, the lower concordance rates observed for conditions such as Barrett's esophagus (25.0%), as shown in Table 2, and neuroendocrine tumors (71.43%), as shown in Table 1, emphasize the variability in endoscopic diagnostic accuracy. These findings are consistent with the existing literature, which has documented the challenges in diagnosing subtle or complex lesions endoscopically.

Correlation between endoscopic findings and histological diagnoses

It varies across different sites of the GIT, with significant correlations observed in the esophagus and stomach but not in the duodenum, indicating variability in diagnostic accuracy [14]. Previous studies have reported moderate to strong correlations in the esophagus, with one study showing a Cohen's kappa value of 0.40, which is higher than the 0.057 observed in the current study, possibly because of differences in sample sizes, diagnostic techniques, and population characteristics [15]. Additionally, the mean age of patients with esophageal lesions aligns with previous findings, with a prevalence in males, supporting the current study's mean age of 55.50 years and 85.7% in males prevalence [14,15]. These discrepancies highlight the importance of considering the various factors that influence diagnostic accuracy at different GIT sites.

The data presented in the tables and supported by previous studies demonstrate a high concordance between endoscopic and histological diagnoses in the esophagus, stomach, and duodenum, indicating the reliability of endoscopic procedures in diagnosing gastrointestinal lesions [14-18]. Specifically, esophageal biopsies showed 100% concordance for various conditions such as Barrett's esophagus, esophageal ulcers, growth, polyps, and ulcers, whereas gastric biopsies exhibited high concordance for erosion, gastric body polyps, and gastric growth. Duodenal biopsies also demonstrated 100% concordance across the different endoscopic findings, highlighting their diagnostic capabilities. Although a highly significant correlation was found between the endoscopic and histological diagnoses of the stomach (p<0.001), the poor agreement indicated by Cohen's kappa statistic (0.000) suggests that, although statistically significant, the practical agreement between the diagnostic methods may be limited.

The challenges in accurately diagnosing duodenal lesions using endoscopy alone are underscored by the lack of significant correlation (p=0.065) and poor agreement (Cohen's kappa=0.027) in the duodenum, as highlighted in research [19]. This contrasts with previous studies that reported significant correlations, emphasizing the necessity for further investigation into diagnostic practices and potential enhancements in endoscopic techniques [20,21]. The diverse spectrum of duodenal lesions, including subepithelial tumors such as neuroendocrine tumors, necessitates a comprehensive approach that integrates imaging techniques such as multidetector CT and MRI to accurately analyze the extent of the disease [19]. Additionally, endoscopic ultrasound-guided fine-needle aspiration has been shown to be safe and accurate for diagnosing duodenal subepithelial lesions, with a reasonably high likelihood of containing malignant tumors, emphasizing the importance of this technique in improving diagnostic outcomes [19,22].

Clinical implications

The results of the present study have several clinical implications. First, the significant age-related increase in upper GIT lesions underscores the need for targeted screening and early intervention strategies for older adults. Gender-specific lifestyle interventions, particularly addressing smoking and alcohol consumption among males, could help mitigate the higher incidence of lesions observed in this group.

Second, the high correlation between endoscopic and histological diagnoses reinforces the role of endoscopy as a primary diagnostic tool for upper GIT lesions. However, variability in concordance rates, particularly for conditions such as gastric ulcers and celiac disease, highlights the need for histopathological confirmation to ensure diagnostic accuracy and appropriate treatment.

Future research directions

Future longitudinal studies should focus on tracking the progression of upper GIT lesions over time, particularly in high-risk groups. Additionally, investigating the genetic and molecular mechanisms underlying observed sex disparities could provide insights into targeted prevention and treatment strategies. Enhancing endoscopic technologies and techniques to improve diagnostic accuracy, especially in conditions with lower concordance rates, should be prioritized.

## Conclusions

This study explored the spectrum of histopathological lesions in the GIT and their correlation with the endoscopic findings. It was found that the age and sex distribution patterns were consistent with previous literature, with a higher prevalence of esophageal lesions in males. Significant correlations were found between endoscopic and histological diagnoses in the esophagus and stomach but not in the duodenum. The agreement between the two diagnostic methods was poor across all sites. These findings highlight the need for improved diagnostic techniques and the integration of multiple methods to enhance the accuracy of GIT lesion diagnosis. Future research should focus on increasing sample sizes, standardizing diagnostic techniques, and conducting focused studies on duodenal lesions to resolve inconsistencies in the correlation findings. Overall, this study underscores the complexity and variability of diagnosing GIT lesions and emphasizes the importance of continuous evaluation and refinement of diagnostic approaches.
